# Structural basis of PI(4,5)P_2_-dependent regulation of GluA1 by phosphatidylinositol-5-phosphate 4-kinase, type II, alpha (PIP5K2A)

**DOI:** 10.1007/s00424-013-1424-8

**Published:** 2014-01-05

**Authors:** Guiscard Seebohm, Eva Wrobel, Michael Pusch, Markus Dicks, Jan Terhag, Veronika Matschke, Ina Rothenberg, Oana N. Ursu, Fabian Hertel, Lutz Pott, Florian Lang, Eric Schulze-Bahr, Michael Hollmann, Raphael Stoll, Nathalie Strutz-Seebohm

**Affiliations:** 1 Institute for Genetics of Heart Diseases (IfGH), —Myocellular Electrophysiology, Department of Cardiovascular Medicine, University Hospital Muenster, 48149 Muenster, Germany; 2 Istituto di Biofisica CNR, 16149 Genova, Italy; 3 Department of Biochemistry II, Biomolecular NMR, Ruhr University Bochum, 44780 Bochum, Germany; 4 Department of Biochemistry I-Receptor Biochemistry and Research Department of Neuroscience, Ruhr University Bochum, 44780 Bochum, Germany; 5 Department of Molecular Pathology, University Tuebingen, 72076 Tuebingen, Germany; 6 Department of Cellular Physiology, Ruhr University Bochum, 44780 Bochum, Germany; 7 Department of Physiology, University Tuebingen, 72076 Tuebingen, Germany; 8 Institute for Genetics of Heart Diseases (IfGH), Department of Cardiovascular Medicine, University Hospital Muenster, 48149 Muenster, Germany

**Keywords:** Glutamate receptor, AMPA receptor, Phosphatidylinositol-4,5-bisphosphate (PIP_2_), PIP_2_ binding, Regulation

## Abstract

Ionotropic glutamate receptors are the most important excitatory receptors in the central nervous system, and their impairment can lead to multiple neuronal diseases. Here, we show that glutamate-induced currents in oocytes expressing GluA1 are increased by coexpression of the schizophrenia-associated phosphoinositide kinase PIP5K2A. This effect was due to enhanced membrane abundance and was blunted by a point mutation (N251S) in PIP5K2A. An increase in GluA1 currents was also observed upon acute injection of PI(4,5)P_2_, the main product of PIP5K2A. By expression of wild-type and mutant PIP5K2A in human embryonic kidney cells, we were able to provide evidence of impaired kinase activity of the mutant PIP5K2A. We defined the region K813–K823 of GluA1 as critical for the PI(4,5)P_2_ effect by performing an alanine scan that suggested PI(4,5)P_2_ binding to this area. A PIP strip assay revealed PI(4,5)P_2_ binding to the C-terminal GluA1 peptide. The present observations disclose a novel mechanism in the regulation of GluA1.

**Electronic supplementary material:**

The online version of this article (doi:10.1007/s00424-013-1424-8) contains supplementary material, which is available to authorized users.

## Introduction

Ionotropic glutamate receptors are the most important mediators of excitatory signal transduction in the central nervous system [35, 36]. They can be pharmacologically classified in three distinct classes: α-amino-3-hydroxy-5-methyl-4-isoxazole propionic acid (AMPA), kainate, and NMDA receptors. The family of the AMPA receptors consists of four subunits, GluA1–4 (GluR1–4, GluRA–D) [41]. The activation of AMPA-type glutamate receptors through the binding of glutamate leads to the activation of a multitude of biochemical pathways in postsynaptic neurons which eventually leads to postsynaptic neuronal plasticity. Changes in synaptic strength can occur by altering the activity and/or abundance of postsynaptic AMPA receptors [19, 31, 42].

Phosphoinositides are phosphorylated derivatives of phosphoinositol and serve as important second messengers in the cell. These phosphorylated lipids are produced at cell membranes during signaling events and contribute to the recruitment and activation of various signaling components as well as the trafficking of intracellular membranes [4, 6]. Phosphoinositides have emerged as major regulators of the binding of cytosolic proteins to the bilayer [43], and there is abundant evidence for the importance of phosphoinositide pathways in synaptic plasticity [14, 22, 26, 30] and ion channel function [16, 18, 27, 39, 44, 45]. However, despite the large body of evidence for the relevance of phosphoinositide pathways for synaptic plasticity, very little is known about the influence of phosphoinositides on glutamate receptor function. The lipid phosphatidylinositol 3,4,5-trisphosphate (PIP_3_) has been shown to control synaptic function by maintaining AMPA receptors clustered at the postsynaptic membrane [2], and for NMDA receptors, it has been suggested that the phosphoinositide 4,5-bisphosphate (PI(4,5)P_2_) might be important for the retention of NMDA receptors at the cell surface of cortical neurons [23]. Phosphorylation of transmembrane AMPA receptor regulatory proteins (TARPs) regulates synaptic AMPA receptors through interaction with membrane lipids [40]. Here, we examine the potential role of the schizophrenia-associated phosphatidylinositol-5-phosphate 4-kinase, type II, alpha (PIP5K2A) in the regulation of AMPA receptors. A schizophrenia association screen identified a point mutation in PIP5K2A (N251S) in schizophrenia patients [32]. The main product of PIP5K2A, PI(4,5)P_2_, is a minor phospholipid component of cell membranes and enriched in the inner layer of the plasma membrane where it is a precursor of the second messengers IP3 and PIP_3_. Moreover, by regulating functions of numerous ion channels and transporters, it is considered a second messenger in its own right. We show a regulatory effect of PI(4,5)P_2_ on GluA1 receptor function, prove direct binding of PI(4,5)P_2_ to a defined amino acid stretch in the GluA1 C-terminus, and analyze the interaction between PI(4,5)P_2_ and GluA1 by an alanine scan, PIP strip assay, and 3D modeling.

## Material and methods

### Mutagenesis, cRNAs, peptides, and lipids

The mutations for the alanine scan (K813A, S814A, R815A, S816A, E817A, S818A, K819A, R820A, M821A, K822A, G823A) were introduced using the QuikChange II Site-Directed Mutagenesis Kit (Agilent Technologies, Waldbronn, Germany) as described in the user manual. The primers used for mutagenesis were the following: ACGCATCCCGTAGCGAGTCGAAGCGG (K813A), TACAAAGCCCGTAGCGAGTCGAAGCGG (S814A), CTACAAATCCGCTAGCGAGTCGAAGCG (R815A), TCCCGTGCCGAGTCGAAGCGGATGAAG (S816A), TCCCGTAGCGCGTCGAAGCGGATGAAG (E817A), TCCCGTAGCGAGGCGAAGCGGATGAAG (S818A), TCCCGTAGCGAGTCGGCGCGGATGAAG (K819A), TCCCGTAGCGAGTCGAAGGCGATGAAG (R820A), TAGCGAGTCGAAGCGGGCGAAGGGT(M821A), AGCGAGTCGAAGCGGATGGCGGGTTTCTG (K822A), ATGAAGGCTTTCTGTTTGATCCCACAGC (G823A). Template complementary DNA (cDNA) was linearized with a suitable restriction enzyme. Complementary RNA (cRNA) was synthesized from 1 μg of linearized cDNA using an in vitro transcription kit (mMessage mMachine T7 Kit, Ambion Ltd., Cambridgeshire, UK). cRNA concentrations were evaluated by spectrophotometry, and transcript quality was checked by agarose gel electrophoresis. PI(4,5)P_2_ diC8 (#P-4508, Echelon Biosciences, Inc., Salt Lake City, USA) was used in the intracellular pipette solution in patch clamp experiments and injection into oocytes, respectively.

### Electrophysiological measurements in *Xenopus* oocytes

Oocytes of stages V–VI were surgically removed from the ovaries of *Xenopus laevis* as described elsewhere [33]. Oocytes were injected with GluA1 cRNA (4 ng/oocyte) or together with PIP5K2A cRNA (4 ng/oocyte) or PIP5K2A-N251S (4 ng/oocyte) using a nanoliter injector 2000 (WPI, Berlin, Germany). For the alanine scan, oocytes were injected with GluA1 cRNA or the respective GluA1 mutant cRNA (8 ng/oocyte). For coinjections of GluA1 and PIP5K2A cRNA, 4 ng/oocyte of each cRNA was used. Standard two-electrode voltage clamp recordings were performed 5–7 days after cRNA injection, employing a TurboTec 10CX amplifier (NPI, Tamm, Germany) and a DA/AD-interface DIGIDATA 1322A (Axon Instruments, CA, USA). Data analyses were done with pClamp/Clampex software (Axon Inc., CA, USA) and Origin 7.0 software (Additive, Friedrichsdorf, Germany). Agonist solutions were prepared in ND-96 buffer (96 mM NaCl, 1.8 mM CaCl_2_, 2.0 mM KCl, 1.0 mM MgCl_2_, and 5 mM hydroxyethyl piperazineethanesulfonic acid (HEPES)–NaOH, pH 7.2 with NaOH, all from Sigma-Aldrich, Munich, Germany).

To rule out calcium influences, 1.8 mM CaCl_2_ was replaced by 1.8 mM MgCl_2_, 1.0 mM BaCl_2_, and 0.3 mM niflumic acid (pH 7.4 with NaOH) in the recording solution, and in addition, oocytes were injected with 1,2-bis(2-aminophenoxy)ethane-*N*,*N*,*N′*,*N′*-tetraacetic acid (BAPTA; 20 nl of 50 mM BAPTA) 60 min prior to recording. Current and voltage electrodes were filled with 3 M KCl and had resistances of 0.5–1.5 MΩ. Oocytes were held at −70 mV, and an agonist (300 μM glutamate, 100 μM cyclothiazide (Tocris, Cologne, Germany)) was applied by superfusion for 10 s at a flow rate of 10–14 ml/min. Half maximal effective concentration (EC_50_) values for glutamate were measured with nine different agonist concentrations. Data from each oocyte were fitted separately with the GraphPad Prism program to obtain an EC_50_ value. EC_50_ values of five to six oocytes were eventually averaged.

### Outside-out current measurements in HEK293 cells

Human embryonic kidney (HEK) 293-T cells were transfected with GluA1(Q)flip-pIRES2-EGFP using Metafectene Pro (Biontex, Martiensried, Germany). One day after transfection, cells were plated onto 30-mm glass bottom dishes (FluoroDish, World Precision Instruments, Sarasota, FL, USA) at a density of 60,000–80,000 cells per dish. Outside-out recordings were performed 2 days after plating using an EPC-9 amplifier (HEKA Elektronik, Lambrecht, Germany). Currents were digitized with a sampling rate of 10 kHz and filtered at 3 kHz. Pipettes were pulled from borosilicate glass to resistances of 10–15 MΩ. The extracellular solution contained 140 mM NaCl, 4 mM KCl, 2 mM CaCl_2_, 1 mM MgCl_2_, and 10 mM HEPES adjusted to pH 7.3 with NaOH. The pipette solution contained 130 mM CsF, 2 mM MgCl_2_, 1 mM CaCl_2_, 11 mM EGTA, and 10 mM HEPES adjusted to pH 7.3 with KOH; PI(4,5)P_2_ diC8 was added to the pipette solution to a final concentration of 20 nM. Brief (10 ms), repetitive (10–100) pulses of glutamate (1 mM) were applied using a borosilicate theta capillary mounted to a piezo-driven actuator (PI Physik Instrumente, Karlsruhe, Germany). Capacitances of the excised patches were determined using the time-domain technique [20]. Nonstationary noise analysis was performed as described in the literature [11, 37]. For data analysis, the freely available software ANA (M. Pusch) was used. Statistical analyses were performed using Prism 5.0 (GraphPad Software, San Diego, CA, USA).

### Relative abundance of EGFP-tagged GluA1 expression in HEK293 cells determined by fluorescence microscopy

HEK293 cells were transfected with plasmid DNA (1.5 μg GluA1/pEGFP; 1.36 μg PIP5K2A/pcDNA3 or PIP5K2A(N251S)/pcDNA3) using Metafectene Pro (Biontex, Martinsried, Germany) and after 24 h seeded at a density of 4 × 10^4^ cells/3.5-cm dish. After another 24 h, the cells were fixed. For fixation, the 3.5-cm dish plate was washed with 1× phosphate-buffered saline (PBS) to remove media and treated with 4 % PFA (Sigma-Aldrich, Taufkirchen, Germany)/PBS for 15 min. Finally, slides were mounted with Fluoromount (Sigma-Aldrich, Taufkirchen, Germany), and samples were examined by fluorescence microscopy (Zeiss Axio ObserverZ.1, Oberkochen, Germany) with the appropriate emission band-pass filters for detection of green fluorescent protein (GFP) and AxioVision 4.8 software. For analysis, ImageJ software was used.

### Determination of intracellular levels of PIP kinases and membrane expression of GluA1 by Western blot

HEK293 cells were maintained in DMEM (Invitrogen, Darmstadt, Germany) supplemented with 10 % fetal bovine serum (Invitrogen) and 1× non-essential amino acids (Sigma-Aldrich, Munich, Germany). To determine expression levels of wild-type and mutant PIP5K2A kinases, cells were plated onto 3.5-cm dishes at a density of 2.5 × 10^5^ cells/dish, grown for 48 h, and then transfected with 3 μg plasmid DNA (PIP5K2A/pcDNA3 or PIP5K2A(N251S)/pcDNA3) using Metafectene Pro (Biontex, Martinsried, Germany). Control cells were treated with Metafectene Pro alone. For lysis of transfected HEK293 cells, the 3.5-cm dish plate was washed with 1× PBS to remove residual media. Then, 150 μl of 1× cell lysis buffer (# 9803, Cell Signaling Technology, Danvers, MA, USA)/3.5-cm dish was added and the plate incubated on ice for 5 min. The cells were scraped and sonicated briefly, followed by a centrifugation for 10 min at 14,000×*g* in a cold microfuge. The supernatant was removed and stored at −80 °C until needed. Prior to gel electrophoresis and Western blotting, a protein assay (DC Protein Assay, Bio-Rad Laboratories, Munich, Germany) was performed to determine the total protein concentrations. For Western blotting, 10 μg of total protein was applied per lane. PIP5K2A monoclonal purified rabbit antibody was used as the primary antibody (1:1000, #ab109128, Abcam, Cambridge, UK). For the detection of α-calnexin, primary rabbit α-calnexin antibody (1:500, sc-11397, Santa Cruz, CA, USA) was used. The secondary antibody used was horseradish peroxidase-conjugated sheep anti-rabbit antibody (1:10,000 dilution, Amersham Bioscience, Freiburg, Germany).

### Cell surface protein isolation

Cell surface protein was isolated according to the manual of Pierce Cell Surface Protein Isolation Kit from Thermo Scientific, Bonn, Germany (Cat# 89881). Briefly, four 75-cm^2^ T75 flasks of HEK293-T cells ((1) without DNA, (2) GluA1/pIRES2/EGFP, (3) GluA1/pIRES2/EGFP + PIP5K2A/pcDNA3, or (4) GluA1/pIRES2/EGFP + PIP5K2A(N251S)/pcDNA3) were transfected with or without the corresponding DNA using the calcium phosphate transfection method. Confluent cells (90–95 %) were incubated with Sulfo-NHS-SS-Biotin for 30 min at 4 °C. Cells were scraped and transferred to a single 50-ml tube after adding a quenching solution to each flask. Cells were centrifuged (4 °C, 500×*g*, 5 min) and transferred into a lysis solution. After sonication, cells were incubated for 30 min on ice, vortexing every 5 min. Cell lysate was centrifuged (4 °C, 10,000×*g*, 2 min); clarified supernatant was transferred to a prepared NeutrAvidin agarose column and incubated for 60 min at room temperature. After washing of the column, it was incubated with SDS-PAGE sample buffer containing DTT for 60 min at room temperature. Elution of the purified cell surface proteins was performed by centrifugation (4 °C, 1,000×*g*, 2 min). Samples were analyzed by Western blot. Antibodies were obtained from Abcam, Cambridge, UK (GluA1, #ab31232, β-Na^+^/K^+^-ATPase, #ab2873).

### Homogenization of HEK293 cell phospholipid extraction and PI(4,5)P_2_ determination by ELISA

Lipid extraction from transfected HEK293 cells was performed as previously described [8]. Briefly, the medium was aspirated from the culture dish, 1 ml ice-cold 0.5 M TCA was added, and the cells were scraped after incubation on ice for 5 min. The cell suspension was centrifuged at 1,500 rpm for 5 min and the pellet washed twice with 1 ml of 5 % TCA + 1 mM EDTA. To remove neutral lipids, 1 ml of methanol/chloroform (2:1) was added, and the mixture was vortexed three times for approximately 30 s over a period of 10 min at room temperature, followed by centrifugation at 1,500 rpm for 5 min. Acidic lipids were extracted from the pellet with 750 μl of methanol/chloroform/12 M HCl (80:40:1) by vortexing four times over a period of 15 min at room temperature. After addition of 250 μl of chloroform and 450 μl of 0.1 M HCl, samples were vortexed vigorously and centrifuged at 1,500 rpm for 5 min. Finally, the lower organic phase was transferred into a new tube and dried in a vacuum centrifuge. Determination of cellular PI(4,5)P_2_ amounts was carried out using the PI(4,5)P_2_ Mass ELISA Kit as specified by the user manual (Echelon Bioscience Inc., Salt Lake City, UT, USA). Briefly, dried lipid extracts were dissolved in 200–600 μl of the sample buffer (PBS, 0.25 % protein stabilizer), loaded into a 96-well plate, and incubated with a PI(4,5)P_2_ detector protein. After incubation, samples were transferred to a PI(4,5)P_2_-coated 96-well plate for competitive binding. To determine the amount of PI(4,5)P_2_ detector protein binding to the plate, a peroxidase-conjugated secondary detection reagent was added. For visualization, a colorimetric substrate was used, and the absorbance at 450 nm was measured with a microplate reader.

### Homology modeling and molecular dynamics simulation of PIP5K2A and GluA1 structures

Consensus homology models of human PIP5K2A and GluA1 were generated using several individual modeling steps within YASARA Structure v10.1: The PIP5K2A consensus homology model is based on the amino acid sequence Lys21–Thr406, and the GluA1 homology model represents the amino acid sequence Leu13–Gly841. Subsequent refinements of high-resolution models using a CASP-approved protocol were achieved [17]. The Protein Data Bank (PDB) database was searched for known structures with a similar sequence using PSI-BLAST [1] to identify potential modeling templates. The templates were ranked based on the alignment score and the structural quality according to WHAT_CHECK [13] obtained from the PDBFinder2 database [12]. PIP5K2A models were built for the top-scoring templates (PIP5K2β: 1bo1 and PIP5K2γ: 2gk9). For both PIP5K2A templates and the GluA1 template (GluA2: 3kg2), the alignment with the target sequence was iteratively optimized using the evolutionary information contained in related sequences (SwissProt and TrEMBL), the structural information contained in the template, and the predicted target secondary structure [15] to obtain a structure-based alignment correction which is partly based on SSALN scoring matrices [28]. An indexed version of the PDB was used to determine the optimal loop anchor points and collect possible loop conformations if insertions and deletions and dead-end eliminations were used to find initial rotamer solutions in the context of a simple repulsive energy function [5]. The loops were optimized by trying hundreds of different conformations, reoptimizing the side chains for all of them. Fine-tuning of side-chain rotamers was performed considering electrostatic and knowledge-based packing interactions as well as solvation effects. Using the AMBER03 force field, an unrestrained high-resolution refinement in explicit solvent was performed. Final hybrid models were built; bad regions in the top-scoring models were iteratively replaced with corresponding fragments from other PIP5K2A models. As GluA1 had only one template, no consensus homology model could be generated. This model is completely based on the solved GluA2 structure [38] (3kg2.pdb). The resulting consensus PIP5K2A homology model and the GluA1 homology model were solved in 0.9 % NaCl solution, and standard molecular dynamics (MD) simulations were run to relax the model using YASARA Structure v10.1. The quality of both PIP5K2A and GluA1 was estimated, and the data are shown in supplemental Fig. S1 and S2 (Online Resource), respectively.

There is a solved crystal structure of PIP5K2A (2YBX deposited in the RCSB protein database by Tresaugues L et al., http://www.rcsb.org/pdb/explore.do?structureId=2YBX), but it lacks three highly relevant regions for our modeling (residues 126–130, 294–325, 367–385). Two of these regions (residues 126–130, 294–325) are directly involved in the formation of the ligand-binding domains. Therefore, template structures 1bo1 and 2gk9 from the highly homologous PIP5K2β/PIP5K2γ represent far better templates to generate a consensus working model of PIP5K2A. However, comparison of the modeled structure to the solved parts of the structure of PIP5K2A over 284 analyzed residues revealed a relatively small root-mean-square deviation (RMSD) of 1.87 ± 1.13 Å, further indicating the relatively high quality of the PIP5K2A model.

### Preparation and conduction of PIP5K2A MD simulations

ATP/ADP were docked into the ATP binding site of the PIP5K2A wild-type (wt) homology model in a similar position as described for PKA in a procedure described before [29]. Both models were energy-minimized, and PI(4,5)P_2_ and PI(5)P were docked to PIP5K2A-ADP and PIP5K2A-ATP, respectively, resulting in the complexes PIP5K2A-ADP–PI(4,5)P_2_ and PIP5K2A-ATP–PI(5)P. These models were energy-minimized again. In both model complexes, the residue Asn251 was mutated to serine to obtain PIP5K2A(N251S)-ADP–PI(4,5)P_2_ and PIP5K2A(N251S)-ATP–PI(5)P. All four models were incorporated in simulation boxes filled with 0.9 % NaCl–H_2_O. MD simulations were performed for 2 ns to drive the models into stable conformations. The following settings were used: force field AMBER03, temperature at 298 K, pressure at 1 bar, pH 7.0, coulomb electrostatics at a cutoff of 7.86, 0.9 % NaCl, solvent density 0.997, 1-fs time steps. The average structures were determined from the 2,000-ps simulations and were used to calculate the root-mean-square fluctuations (RMSFs) for the respective PIP5K2A model (supplemental Fig. 3, Online Resource). Furthermore, RMSD and energies were computed. Contacts between ligands and the receptor site in PIP5K2A wt and N251S were determined using YASARA Structure v10.1. Superimposed figures of model pairs are shown in supplemental Fig. 4 (Online Resource).

### Preparation and conduction of membrane simulations of partial GluA1

The GluA1 model generated was elongated at the C-terminal ends to Gly841 assuming an α-helical structure of the residues as predicted (supplemental Fig. 2a, Online Resource). The generated GluA1 homology model comprises 828 residues (Leu13–Gly841) from four subunits. The total of 3,312 amino acids represents a relatively large protein complex on which all-atoms-mobile simulations in membranes are difficult. As the N-terminal domain (NTD) is not expected to significantly influence the inner pore domain, the NTD residues (Leu13–Tyr409) were removed. The resulting GluA1 model comprised only 433 residues (the tetramer has 1,732 amino acids). Four PI(4,5)P_2_ molecules were generated de novo using ACD/ChemSketch (version 12.01), manually docked in close proximity to the residues Ser813–Lys822, and the resultant GluA1–PI(4,5)P_2_ complex was energy-minimized. The GluA1 model without PI(4,5)P_2_ and the GluA1–PI(4,5)P_2_ complex were both placed into a simulation box and inserted into a virtual membrane consisting of equal quantities of phosphatidylethanolamine (PEA), phosphatidylcholine (PCH), and phosphatidylserine (PSE; PEA/PCH/PSE—33 %:33 %:33 %). Membrane height was 43 Å (density ∼0.861 g/l), and the height of the hydrophobic membrane core was 28 Å. The GluA1 model without PI(4,5)P_2_ and the GluA1–PI(4,5)P_2_ complex were shrunk to 50 % of their size, and lipids bumping into the shrunk protein were deleted. Stepwise, the size of the protein/PI(4,5)P_2_ complexes was expanded to reach their regular size and to fill the membrane pore. Each expansion step was followed by energy minimizations. The simulation box was filled with water, and 0.9 % NaCl was added to the system. MD simulations (2 ns) were performed on GluA1 models in complex with or without PI(4,5)P_2_. The following simulation settings were used for the molecular dynamics simulation: force field AMBER03, temperature at 298 K, pressure at 1 bar, pH 7.0, coulomb electrostatics at a cutoff of 7.86, 0.9 % NaCl, solvent density 0.997, pH 7.0, 1-fs time steps, periodic boundaries, all atoms mobile. The average structures were determined from the simulations and were used to calculate the RMSFs for the respective model. Further, RMSD and energies were computed. During a 25-ps initial equilibration period, the membrane was artificially stabilized so that it could repack and cover the solute, while solvent water molecules are kept outside the membrane core for the initial 250 ps of the simulation.

### PIP strip assay

PIP strip membranes facilitate the analysis of phosphoinositide protein interactions by protein–lipid overlay assay. PIP strip membranes from Life Technologies (Darmstadt, Germany) were used (# P23750). GluA1 peptide was synthesized by Caslo (Lyngby, Denmark), and a fluorescein isothiocyanate (FITC) fluorochrome was conjugated to the N-terminus via an aminohexanoic acid (Ahx) linker. The GluA1 peptide sequence is (FITC)-Ahx-GALIEFCYKSRSESKRMKG. The first glycine was introduced for more flexibility of the peptide. The assay was performed as follows: the strip membrane was blocked with TBS-T + 3 % fatty-acid-free bovine serum albumin (BSA) (TBS-T = 10 mM Tris–HCl, pH 8.0; 150 mM NaCl; 0.1 % (*v*/*v*) Tween 20) and gently agitated for 1 h at room temperature. The peptide was solved in TBS-T + 3 % fatty-acid-free BSA and applied to the membrane. The membrane was incubated with 50 μg/ml peptide for 1 h at room temperature by gentle agitation. The washing of the membrane was performed three times with TBS-T + 3 % fatty-acid-free BSA for 10 min each time. For detection of fluorescence signal, the Molecular Imager^TM^ GelDoc from BioRad (Munich, Germany) was used. All incubation steps were performed in the dark.

### Statistical analysis

Statistical analyses of the data were performed with Origin 7.0. Student’s *t* test for unpaired data and ANOVA was applied, and *p* < 0.05 was considered statistically significant. Oocyte and HEK293 cell experiments were analyzed using Student’s *t* test, ANOVA, or Mann–Whitney test, as applicable.

## Results

### Regulation of GluA1 by PIP5K2A

**Fig. 1:**
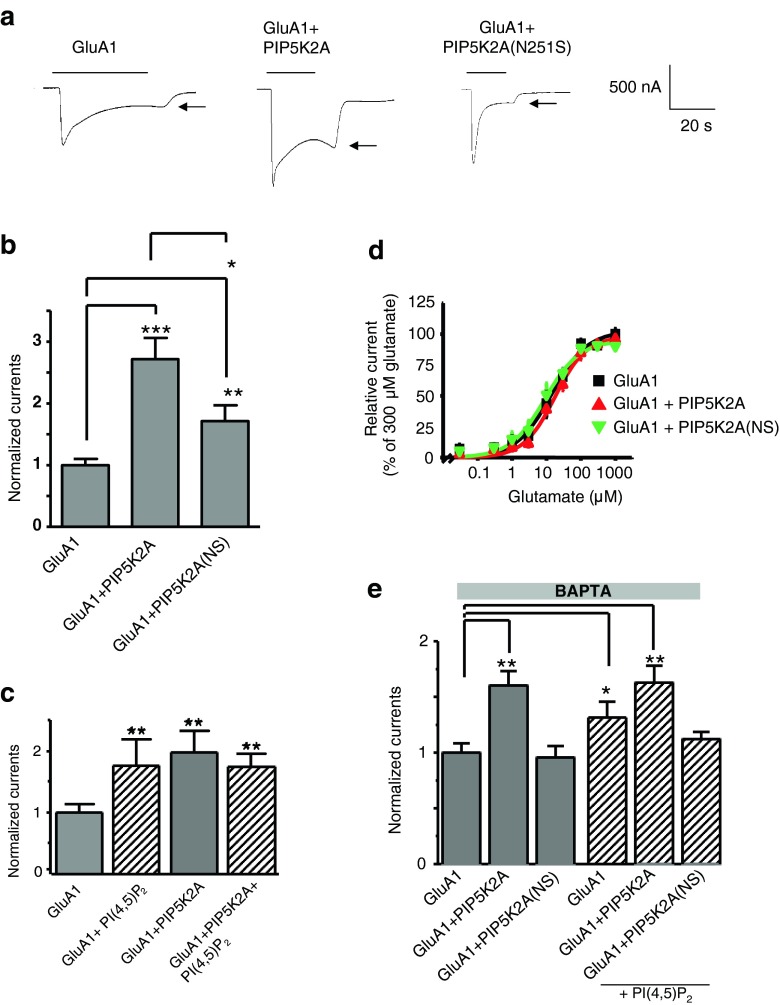
Effects of PIP5K2A or PI(4,5)P_2_ but not of PIP5K2A(N251S) on properties of GluA1 currents in oocytes. **a** Representative current traces measured in *Xenopus* oocytes in response to superfusion with 300 μM glutamate and 100 μM cyclothiazide. All currents were measured at −70 mV. Steady-state currents (indicated by *arrows*) were used for analysis. **b** GluA1 current amplitudes in oocytes expressing GluA1, GluA1 + PIP5K2A, or GluA1 + PIP5K2A(N251S) normalized to the GluA1 currents. Numbers of oocytes vary between 23 and 35, and significant changes are indicated by ***p < 0.001, **p < 0.01, or *p < 0.05. **c** GluA1 current amplitudes in oocytes expressing GluA1 or GluA1 + PIP5K2A are shown. For *bars 2* and *4*, PI(4,5)P_2_ was acutely injected 5 min prior to measurements. Numbers of oocytes varied between 12 and 17, and significant changes are indicated by **p < 0.01. *Striped bars* indicate results after injections of oocytes with water-soluble PI(4,5)P_2_. **d** Concentration–response curves for glutamate of GluA1, GluA1 + PIP5K2A, and GluA1 + PIP5K2A(N251S) expressed in oocytes. Mean values ± SEM were calculated from independent measurements (*n* = 5–6). **e** Oocytes were injected with BAPTA prior to the experiments. GluA1 current amplitudes were determined in oocytes expressing GluA1 or GluA1 + PIP5K2A, without or after acute injection with a water-soluble PI(4,5)P_2_ analog. Numbers of oocytes varied between 17 and 26, and significant changes are indicated by **p < 0.01 or *p < 0.05. *Striped bars* indicate results from oocytes injected with water-soluble PI(4,5)P_2_ prior to recording

To test for regulation of GluA1 by PIP5K2A kinase, we heterologously expressed the AMPA receptor subunit GluA1 in *Xenopus* oocytes alone or together with PIP5K2A or PIP5K2A(N251S). The point mutation at position 251 in PIP5K2A(N251S) was identified by Schwab and colleagues in schizophrenia patients when performing a schizophrenia association screen [32]. As illustrated in Fig. 1, the glutamate-induced currents were significantly larger in *Xenopus* oocytes overexpressing GluA1 together with PIP5K2A than in oocytes expressing GluA1 alone. The stimulating effect of PIP5K2A on GluA1 was significantly less pronounced when GluA1 was coexpressed with the PIP5K2A (N251S) mutant (Fig. 1a, b).

### Effect of PI(4,5)P_2_ on electrophysiological features of GluA1

PI(4,5)P_2_ is the main product of PIP5K2A. To clarify whether stimulation of GluA1 by PIP5K2A results from an effect of the lipid product PI(4,5)P_2_ or another effect of the enzyme, we injected a water-soluble form of PI(4,5)P_2_ and determined GluA1 currents. As illustrated in Fig. 1c, injection of PI(4,5)P_2_ before recording caused a significant increase in GluA1 current amplitudes. This stimulation by PI(4,5)P_2_ was not further increased by coexpression of the kinase PIP5K2A (Fig. 1c), suggesting that PIP5K2A and the major product of PIP5K2A, PI(4,5)P_2_, act in the same cascade. The PIP5K2A-dependent increase in GluA1 current amplitudes could be due to enhanced membrane abundance of GluA1 subunits and/or a consequence of changed receptor properties. Analysis of the EC_50_ values (glutamate) for GluA1 (17.2 ± 4.5 μM) or for GluA1 with PIP5K2A (19.6 ± 2.3 μM) or PIP5K2A(N251S) (10.6 ± 3.6 μM) revealed no significant differences when tested in oocytes (Fig. 1d). In order to test if the effect in oocytes was due to activation of calcium-activated chloride channels endogenous in oocytes, CaCl_2_ in the extracellular solution was replaced by MgCl_2_ and oocytes were injected with the calcium chelator BAPTA to buffer cytosolic calcium and to prevent activation of endogenous calcium-activated channels. Under these conditions, the PIP5K2A kinases and the water-soluble PI(4,5)P_2_ diC8 caused similar albeit slightly smaller but still significant effects on GluA1 channel functions (Fig. 1e).

**Fig. 2:**
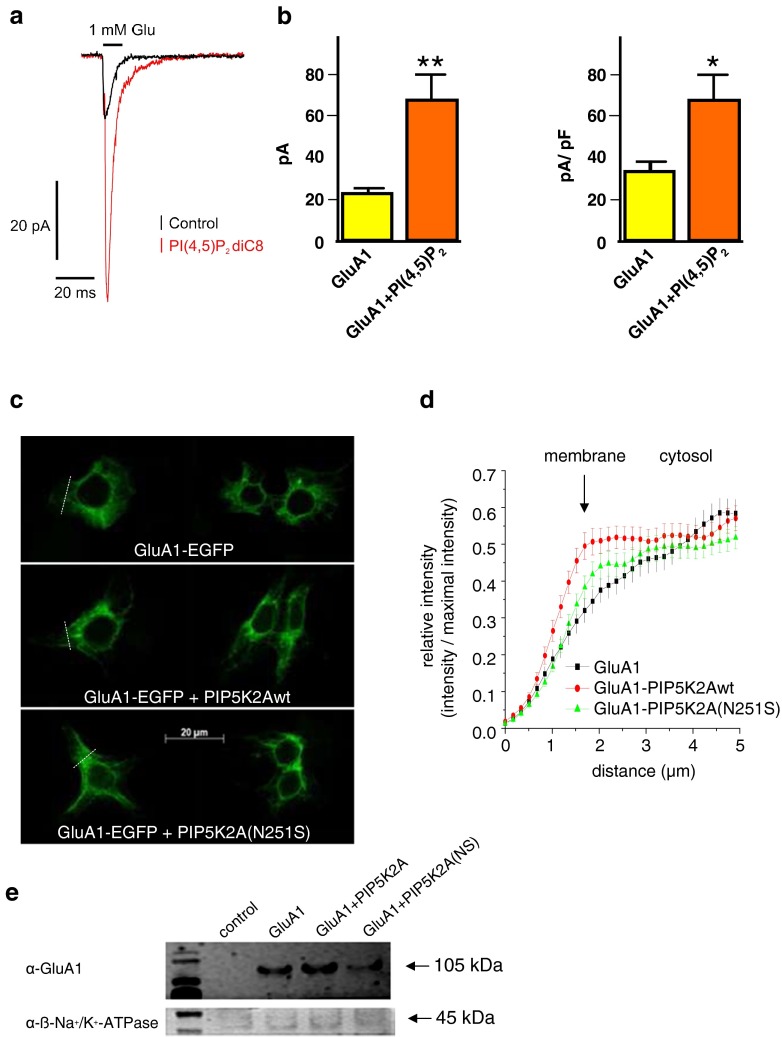
Effect of PI(4,5)P_2_ on properties of GluA1 currents and of PIP5K2A on GluA1 plasma membrane expression. **a** Overlay of GluA1 currents in the absence (*black trace*) and presence (*red trace*) of PI(4,5)P_2_ diC8; traces represent averages of 10–20 repeated applications. **b** Mean peak amplitudes (±SEM) of peak currents evoked by 10-ms application of 1 mM glutamate are shown. Under control conditions, the mean peak current amplitude was 22 ± 4 pA. When PI(4,5)P_2_ diC8 was present in the pipette solution, the mean peak current was significantly larger (70 ± 11 pA, **p = 0.0009, *n* = 10, Mann–Whitney test). To compensate for patches of different sizes, current densities were calculated (control: 32 ± 6 pA/pF, PI(4,5)P_2_ diC8: 66 ± 13 pA/pF; *p = 0.0019, *n* = 10, Mann–Whitney test). **c** Representative pictures taken from HEK293 cells transfected with GluA1-EGFP alone or GluA1-EGFP together with the kinases PIP5K2A or PIP5K2A(N251S). *Dashed lines* indicate the position of intensity analysis. **d** Analysis of GFP fluorescence intensity at the plasma membrane. Line plots were analyzed and the mean data ± SEM is shown. The estimated position of the membrane (*arrow*) and the side of the cytosol are indicated. Number of cells analyzed: GluA1: *n* = 53, GluA1 + PIP5K2Awt: *n* = 55, GluA1 + PIP5K2A(N251S): *n* = 66. **e** Western blot of membrane GluA1 expression level in HEK293 cells after coexpression with either PIP5K2A or PIP5K2A(N251S) or GluA1 expressed alone. As control, only transfection reagent was transfected (lane 1). β-Na^+^/K^+^-ATPase was used as control protein

In order to analyze fast kinetic properties of the receptor, we applied the outside-out patch clamp technique to HEK293 cells transiently transfected with GluA1. In this system, it was possible to observe effects on GluA1 current amplitudes and GluA1 kinetics upon application of PI(4,5)P_2_ diC8 via the recording pipette. Significant changes in current amplitude were found for GluA1-expressing HEK293 cells exposed to PI(4,5)P_2_ diC8 compared to cells not exposed to the lipid (Fig. 2a, b). Single-channel properties were analyzed by non-stationary noise analysis and showed no significant differences (data not shown). The macroscopic kinetics was not significantly altered by the PIP_2_ analog in the pipette (supplemental Fig. S5, Online Resource).

To test for membrane surface GluA1 protein, the relative abundance of an EGFP-tagged GluA1 expressed alone or together with PIP5K2A variants was analyzed. GluA1–EGFP abundance was increased in HEK293 cells coexpressed with PIP5K2A wt compared to GluA1–EGFP expressed without additional kinase variants (Fig. 2c, d). Coexpression of GluA1 with PIP5K2A(N251S) resulted in an increase in GluA1 abundance, although not as much as coexpression with PIP5K2A did. Cell surface protein isolation also revealed an increase of membrane GluA1 after coexpression with PIP5K2A in HEK293 cells, although a reduction of GluA1 expression upon coexpression with PIP5K2A(N251S) compared to GluA1 alone was observed (Fig. 2e). Therefore, enhanced GluA1 plasma membrane expression as a result of activation of the PIP5K2A–PI(4,5)P_2_ cascade may be the reason for the increase in GluA1 function.

### PI(4,5)P_2_ assay for PIP5K2A and PIP5K2A(N251S)

**Fig. 3:**
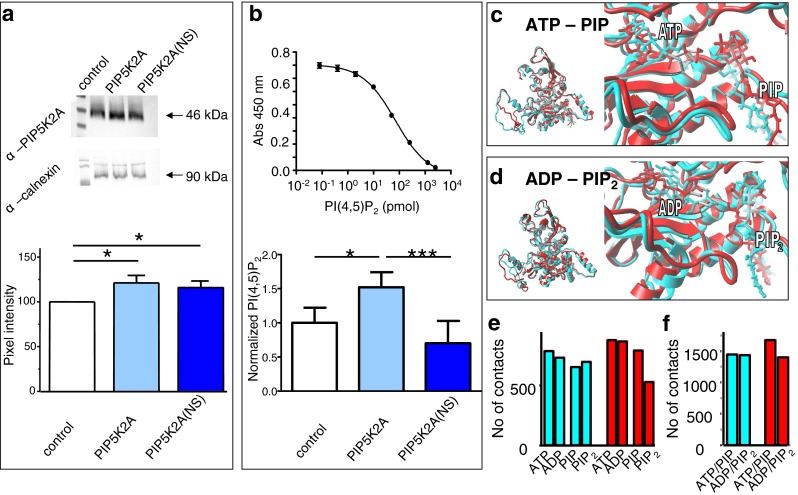
Reduced PI(4,5)P_2_ production by PIP5K2A(N251S) could be a result of structural changes at the catalytic site of the mutant. **a** Representative Western blot of either mock-transfected HEK293 cells or HEK293 cells transfected with PIP5K2A or PIP5K2A(N251S) cDNA. α-Calnexin was used as a marker for proper loading. Bar graph showing the total protein abundance of PIP5K2A protein of mock-transfected HEK293 cells and HEK293 cells transfected with PIP5K2A or PIP5K2A(N251S) cDNA. The band intensity was quantified by densitometry. Number of experiments used for analysis, *n* = 7. **b** ELISA assay for detection of PI(4,5)P_2_ in HEK293 cells expressing either PIP5K2A or PIP5K2A(N251S). Standard curve shows absorbance values obtained versus amount of standard PI(4,5)P_2_ concentrations. Below, graph shows concentrations of PI(4,5)P_2_ in mock-transfected cells in comparison to cells expressing either PIP5K2A or PIP5K2A(N251S). Significant change is indicated by ***p < 0.001 or *p < 0.05 (*n* = 6–7). **c**–**f** Molecular dynamics simulations of PIP5K2A wt and PIP5K2A(N251S). **c**, **d** A PIP5K2A wt homology model is depicted. ATP/ADP, PI(4,5)P_2_, and PI(5)P were docked into the ATP binding site of the PIP5K2A wt (*turquoise*) and mutant homology model (*red*) in a similar position as proposed for the related kinase PIP5K2B, resulting in the complexes PIP5K2A-ADP-PI(4,5)P_2_, PIP5K2A-ATP-PI(5)P, PIP5K2A(N251S)-ADP-PI(4,5)P_2_, and PIP5K2A(N251S)-ATP-PI(5)P. MD simulations covering 2,000 ps were performed on all four models. Overlays of the final structures are shown in **c** and **d. e**, **f** The affinity of a ligand to its receptor increases with increasing numbers of atom contacts as a result of increased van der Waals interactions. The number of contacts of the respective ligands ATP/ADP and PI(5)P/PI(4,5)P_2_ with their receptors in PIP5K2A wt (*red*) and PIP5K2A(N251S) (*turquoise*) were determined. The combined interactions of both ligands with the respective kinase determine the stability of the enzyme-ligand complexes. The combined number of contacts of both ligands with the kinase receptors is shown in **f**. The marked differences in contacts between ATP/PI(5)P and ADP/PI(4,5)P_2_ indicate an increased interaction and thus an increased affinity for ATP/PI(5)P

The impact of the schizophrenia-associated mutation N251S on the function of PIP5K2A thus far has not been investigated. The main catalytic function of PIP5K2A is the phosphorylation of PI(5)P to produce PI(4,5)P_2_. To test whether the mutation N251S affects PIP5K2A function, we performed a quantitative PI(4,5)P_2_ assay. After the treatment of HEK293 cells with transfection reagent plus PIP5K2A or PIP5K2A(N251S) cDNA or with transfection reagent alone, we first verified the overexpression of both PIP5K2A variants and the comparability of their expression levels via Western blot (Fig. 3a). Mock-transfected HEK293 cells as well as HEK293 cells transfected with either PIP5K2A or PIP5K2A(N251S) were lysed and the lipids isolated. Then, a specific PI(4,5)P_2_ ELISA was performed to compare PI(4,5)P_2_ production by PIP5K2A or PIP5K2A(N251S). As shown in Fig. 3b, HEK293 cells overexpressing PIP5K2A show a significant increase in PI(4,5)P_2_ amounts compared to mock-transfected cells. In contrast, the PI(4,5)P_2_ amounts of HEK293 cells expressing the mutant kinase PIP5K2A(N251S) were twofold lower compared to cells expressing wild-type PIP5K2A and on the same level as mock-transfected cells, supporting the notion that the mutation N251S results in reduced kinase activity.

### Molecular simulations of PIP5K2A wt and PIP5K2A(N251S)

We constructed a PIP5K2A homology model to visualize the catalytic domain of PIP5K2A and the location of the N251S mutation in PIP5K2A (Fig. 3c–f). The model was based on phosphatidylinositol phosphate kinase, type II, beta (PIP5K2β) (1bo1A and 2gk9). PIP5K2A residues 29 to 406 could be modeled on ∼70 % sequence identity to PIP5K2β and PIP5K2γ. ATP/ADP, PI(5)P, and PI(4,5)P_2_ were docked into the ATP binding site of the PIP5K2A wt homology model in a similar position as proposed for PIP5K2β [29], resulting in the complexes PIP5K2A-ATP–PI(5)P (Fig. 3c) and PIP5K2A-ADP–PI(4,5)P_2_ (Fig. 3d). In both model complexes, the residue Asn251 was mutated to serine to obtain PIP5K2A(N251S)-ADP–PI(4,5)P_2_ and PIP5K2A(N251S)-ATP–PI(5)P. On all four models, 2-ns MD simulations were performed. Overlays of the final structures are shown in Fig. 3c, d. Particular differences can be observed in the PIP5K2A(N251S)-ATP–PI(5)P complex as a result of the mutation (see also supplemental Fig. S3–S4, Online Resource). In order to gain insights into the mechanism of reduced catalytic activity of PIP5K2A(N251S), we analyzed the number of atomic contacts. It can be assumed that the larger the number of contact points between a receptor and its ligand, the stronger the interaction energy and the more stable the ligand–receptor complex. Thus, enzyme–ligand complexes similarly should have many contact points (and thus interaction energies) to avoid energetic barriers during a catalytic cycle. Our simulations imply that this holds true for the PIP5K2A wt ADP–PI(4,5)P_2_ and PIP5K2A wt ATP–PI(5)P but not for the PIP5K2A(N251S)-ADP–PI(4,5)P_2_ and PIP5K2A(N251S)-ATP–PI(5)P complexes. The PIP5K2A(N251S)-ATP–PI(5)P complex has more contact points than the PIP5K2A(N251S)-ADP–PI(4,5)P_2_ complex. The former complex can be assumed to be more stable, creating an energy barrier during the catalytic circle and reducing the catalytic activity of PIP5K2A(N251S).

### Definition of the PI(4,5)P_2_-sensitive region in GluA1

**Fig. 4:**
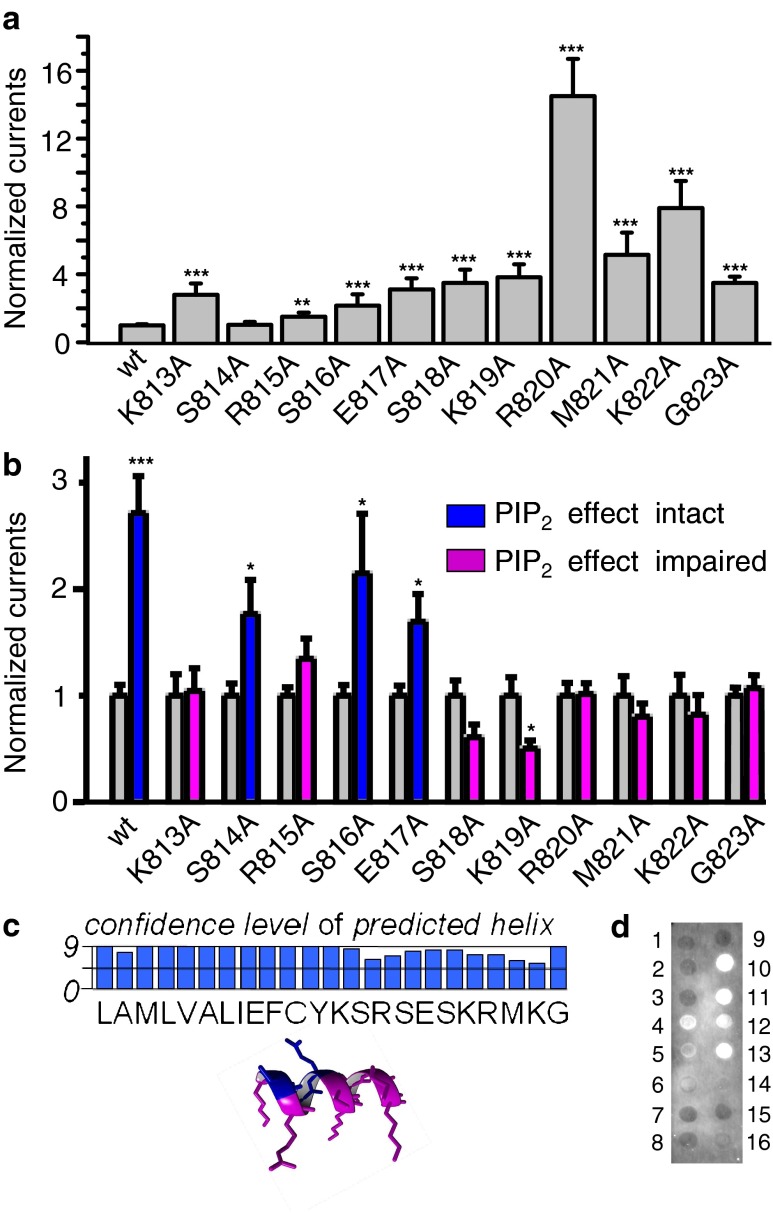
Alanine scan and PIP strip assay reveal the binding of PI(4,5)P_2_ to the C-terminal domain of GluA1. **a** Glutamate + cyclothiazide-evoked current amplitudes in oocytes expressing GluA1 or GluA1 point mutants (K813A through G823A) normalized to the GluA1 currents. Statistically significant current changes of the mutants compared to GluA1 wt are indicated by ***p < 0.005 or **p < 0.01. **b** GluA1 or GluA1 point mutant current amplitudes in oocytes with (*right bars*) or without (*left bars*) coexpression of PIP5K2A. *Blue bars* indicate that the PI(4,5)P_2_ effect is present; *magenta bars* indicate an impaired PI(4,5)P_2_ effect. Numbers of oocytes measured, *n* = 10–35. Statistically significant current changes of GluA1 by PIP5K2A are indicated by ***p < 0.005 or *p < 0.05. **c** Confidence level of a predicted α-helical secondary structure of a GluA1 peptide and 3D model of the region of GluA1 chosen for the alanine scan are shown. The same color code used as for the *bar diagram* in **b**, where *blue* indicates amino acids probably not involved in PI(4,5)P_2_ binding while *magenta* indicates amino acids that may play a role in PI(4,5)P_2_ binding. **d** N-terminal FITC-labeled GluA1 peptide shows binding to PIP, PIP_2_, and PIP_3_. PIP strip membranes were used for the detection of peptide binding to various lipids. *1* = lysophosphatidic acid, *2* = lysophosphatidylcholine, *3* = phosphadidylinositol, *4* = PtdIns(3)P, *5* = PtdIns(4)P, *6* = PtdIns(5)P, *7* = phosphatidylthanolamine, *8* = phosphatidylcholine, *9* = spingosine 1-phosphate, *10* = PtdIns(3,4)P_2_, *11* = PtdIns(3,5)P_2_, *12* = PtdIns(4,5)P_2_, *13* = PtdIns(3,4,5)P_3_, *14* = phosphatidic acid, *15* = phosphatidylserine, *16* = blank

Our data point to an effect of PI(4,5)P_2_ on GluA1. We reasoned that in the case of PI(4,5)P_2_ directly binding to GluA1, the binding region should be located in close proximity to the inner leaflet of the plasma membrane. The C-terminal amino acids K813–G823 span the region closest to the inner leaflet of the membrane. Therefore, we performed an alanine scanning mutagenesis of this stretch of the GluA1 sequence. As shown in Fig. 4a, all GluA1 alanine mutants were functional ion channels, and some even revealed a considerable increase in current amplitudes compared to GluA1 wild type when expressed in *Xenopus* oocytes.

To test for their possible involvement in PI(4,5)P_2_ binding, we coexpressed the GluA1 mutants (K813A–G823A) with PIP5K2A in oocytes and determined maximal current amplitudes. Alanine mutations at positions S814, S816, and E817 showed increased current amplitudes upon coexpression with PIP5K2A. For the other eight mutations (K813A, R815A, S818A, K819A, R820A, M821, K822A, and G823A), the sensitivity of currents to coexpression with PIP5K2A was lost, indicating that those amino acids might be involved in PI(4,5)P_2_ binding (Fig. 4b). The model shown in Fig. 4c depicts the likely orientation of side chains of amino acids possibly involved in PI(4,5)P_2_ binding (magenta) compared to those unlikely to be involved (blue). By using PIP strip membranes, we analyzed the binding of a GluA1 peptide, including the above identified amino acids possibly involved in PI(4,5)P_2_ binding, to phospholipids. The peptide was labeled at the N-terminal with FITC for easy fluorescence detection. The assay proved direct binding of the GluA1 peptide to PI(4,5)P_2_ as well as to PIP, PI(3,5)P_2_, PI(3,4)P_2_, PI(3,4,5)P_3_, and phosphatidic acid (Fig. 4d).

Therefore, these results of the alanine scan and the PIP strip assay clearly suggest the binding of PI(4,5)P_2_ to the C-terminus of GluA1, which is in close proximity of the inner plasma membrane.

### Structure of the GluA1/PI(4,5)P_2_ complex based on homology modeling

**Fig. 5:**
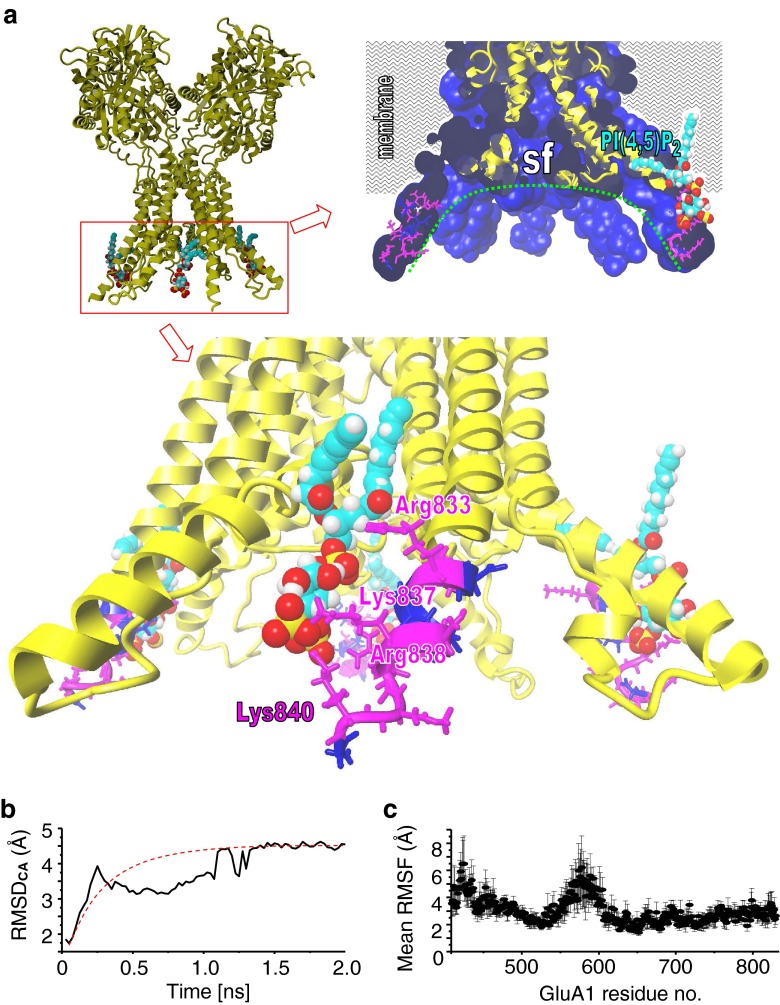
Molecular dynamics simulations of GluA1 alone and in complex with PI(4,5)P_2_. **a** A GluA1 wt homology model was built for residues 410–841. In the case of GluA1 in the presence of 4 PI(4,5)P_2_, one phospholipid per subunit was manually docked in close proximity to the residues (Lys833, Lys837, Arg838, and Lys840) identified in the alanine scan as PI(4,5)P_2_-relevant residues (Fig. 4e). GluA1-PI(4,5)P_2_ was inserted into a PEA/PCH/PSE (each 33 %) membrane. A 2,000-ps MD simulation was performed (details are given in “Material and methods”). The final averaged structure is shown in **a**. A close-up of the partial inner pore with backbone structures (*yellow*) within the surface (*blue*) and cut at the center of the pore is shown in the *upper right figure*. The position of the membrane core is indicated. The inner pore following the selectivity filter (*sf*) forms a bowl-like structure (*green dotted line*). The *lower figure* shows PI(4,5)P_2_ (in CPK colors), and the effective residues and non-effective residues identified in the alanine scan are colored *magenta* and *blue*, respectively. **b** The mean RMSD ± SEM of the complete models compared to the initial structures are shown. **c** The RMSFs of residues compared to the averaged structure are shown

Our GluA1 model represents the residues 433–841 including the C-terminal end, assuming an α-helical structure of these residues, as predicted by the PSIPRED server (Fig. 4c). Four PI(4,5)P_2_ molecules (one PI(4,5)P_2_ for each subunit) were manually docked in close proximity to the residues Ser813–Lys822 (Fig. 5a). The GluA1 model without PI(4,5)P_2_ and the modeled GluA1–PI(4,5)P_2_ complex were inserted into a PEA, PCH, or PSE membrane (33 %:33 %:33 %). MD simulations (2,000 ps) were performed on both models, and mean RMSD calculations suggested that the models had reached a stable conformation during the simulation (Fig. 5b). Calculation of the RMSFs for each model indicated that extracellular regions are flexible whereas membrane-spanning regions and the putative PI(4,5)P_2_-interacting regions (residues 813–823) are rather rigid, consistent with the idea that conformational space of residues in these regions is restricted by the surrounding membrane. As residues 813–823 are positioned outside the membrane core, stabilization may result from interaction with the PI(4,5)P_2_ headgroups. Indeed, stabilizing electrostatic interactions of specifically Lys815, Lys819, Arg820, and Lys822 side chains with the negatively charged PI(4,5)P_2_ head group occur during the simulation and can be observed in the averaged structural models (Fig. 5c).

## Discussion

PI(4,5)P_2_ is the main product of PIP5K2A, a kinase which is associated with schizophrenia [32]. Interestingly, GluA1 has also been implicated in the pathophysiology of schizophrenia [9, 21, 24, 25]. Decreased expression levels of GluA1 have been found in the brain of some schizophrenic patients. GluA1 has also been connected with genetic susceptibility for schizophrenia [21]. However, as mentioned above, schizophrenia is a complex multifaceted disorder, and it has to be considered that under some conditions, an alteration of AMPA receptor conductance within the nervous system may induce other physiological events that induce behaviors that meet the criteria for classification as schizophrenia.

Here, we find that the schizophrenia-associated kinase mutant PIP5K2A(N251S) less effectively produces PI(4,5)P_2_. Homology modeling and molecular dynamics simulation suggest that the mutation has a relatively global effect on PIP5K2A structure and dynamics. As the distances between residue N251 and the ligands are relatively large (Cα-PIP ∼31 Å, Cα-ATP ∼27 Å), the effects on protein structure and function must be of allosteric nature. Possibly, the relatively global effects on the protein structure by the mutation result from destabilization of a loop (residues 215–251) involved in the formation of both ligand domains. The structural and dynamic nature of this loop may determine access of ligands and stability of the ligand–protein complexes as suggested by the MD simulations. As a result of the mutation, an energetic barrier in the catalytic cycle may prevent high-throughput catalyses as suggested by our MD simulations (Fig. 3).

It is known that PI(4,5)P_2_ is a quantitatively minor phospholipid component of cell membranes and enriched at the plasma membrane where it is an important substrate for several signaling proteins, including several ion channels [7]. Here, we show that the stimulating effect of PI(4,5)P_2_ on GluA1 currents is caused by direct binding to the C-terminus of GluA1 rather than a consequence of activation of second messengers by PI(4,5)P_2_. Our 3D molecular model suggests that PI(4,5)P_2_ stimulates GluA1 by interaction with the plasma membrane-adjacent region of the GluA1 C-terminus. A similar effect has recently been shown for the transmembrane AMPA receptor regulatory protein (TARP) molecules [40]. The TARP stargazin appears to interact with negatively charged lipid bilayers in a phosphorylation-dependent manner. In a non-phosphorylated state, TARPs interact with negatively charged lipid bilayers, which inhibit TARP (stargazin) binding to PSD-95. When TARPs are phosphorylated, binding to PSD-95 is possible, resulting in enhancement of synaptic AMPA receptor activity [40]. Our observations allow a similar interpretation: the binding of PI(4,5)P_2_ to the C-terminus of GluA1 could impair binding of regulatory molecules, thus modifying receptor activity by increasing GluA1 protein at the plasma membrane resulting in increased ion channel function by altered clustering. This interpretation is supported by our suggestive experimental data as well as the 3D modeling. Interestingly, GluA1 amino acids shown here to be crucial for PIP_2_/PIP_3_ binding have also been reported to be the sites for phosphorylation, palmitoylation, and Band 4.1 interaction, implying complex cross talk of regulating signals [3, 10]. The group of Esteban identified PIP_3_ as a lipid-controlling synaptic function by maintaining AMPA receptor clustering at the postsynaptic membrane [2]. The mechanism of PIP_3_ action was not examined in that publication. Theoretically, by increasing the pool of diphosphoinositol, the pool of triphosphoinositol could be increased secondarily to enhance GluA1 stimulation. It seems rather likely that both PIP_2_ and PIP_3_ are involved in clustering processes, especially since we show that the identified GluA1 sequence seems to be a promiscuous binding site of PIP, PIP_2_, and PIP_3_. In a previous study, we showed the regulation of GluA1 by PIKfyve, which produces PI(3,5)P_2_ ([34]), the most abundant phospholipid in recycling vesicles. It can be assumed that the locally most abundant phospholipid preferentially binds to the identified GluA1 sequence. Therefore, it may depend on the localization of the receptor which phospholipid binds to it. In recycling vesicles, PI(3,5)P_2_ is the most abundant phospholipid, and at plasma membranes, PI(4,5)_2_ and PI(3,4,5)P_3_ are most abundantly expressed. Additionally, the local concentration of the respective phospholipid species is possibly determined by the locally present kinases. It is reasonable to assume that a functionally impaired kinase like PI5K2A(N251S) may disturb local PIP compositions leading to altered cellular function and possibly diseases including schizophrenia.

In conclusion, the present observations disclose a novel mechanism regulating GluA1 function that may contribute to the pathogenesis of schizophrenia. Furthermore, we show for the first time that PI(4,5)P_2_ directly interacts with the C-terminus of GluA1. Finally, we propose the structure of this carboxyterminal region of GluA1 based on homology modeling.

## Electronic supplementary material

Below is the link to the electronic supplementary material.

ESM 1 (PDF 280 kb)
