# Supplementary material for: Structural basis of PI(4,5)P2-dependent regulation of GluA1 by phosphatidylinositol-5-phosphate 4-kinase, type II, alpha (PIP5K2A)
Source: Pflugers Arch. 2014 Jan 5;466(10):1885–97. doi: 10.1007/s00424-013-1424-8 (PMC4159565; doi:10.1007/s00424-013-1424-8)
Supplement: Supplementary file 1 — (PDF 280 kb) [file 424_2013_1424_MOESM1_ESM.pdf]

Supplementary Material

Pflügers Archive- European Journal of Physiology

Structural Basis of PI(4,5)P_2-Dependent Modulation of GluA1 by Phosphatidylinositol-5-Phosphate 4-Kinase, Type II, Alpha (PIP5K2A)

Guiscard Seebohm, Eva Wrobel, Michael Pusch, Markus Dicks, Jan Terhag, Veronika Matschke, Ina Rothenberg, Nicoleta-Oana Ursu, Fabian Hertel, Lutz Pott, Florian Lang, Eric Schulze-Bahr, Michael Hollmann, Raphael Stoll, Nathalie Strutz-Seebohm

To whom correspondence should be addressed:
Nathalie Strutz-Seebohm, Institute for Genetics of Heart Diseases (IfGH) – Myocellular Electrophysiology, Department of Cardiovascular Medicine, University Hospital Muenster, D-48149 Münster, Germany, Tel: +49 (0)251/83-58255, Fax: +49 (0)251/83-58257, E-mail: Nathalie.Strutz-Seebohm@ukmuenster.de

Supplemental Fig 1. Quality of the PIP5K2A homology model. The final PIP5K2A homology model was subjected to a final round of simulated annealing minimization in explicit solvent in YASARA Structure *v*10.1. The quality of the PIP5K2A homology consensus model was estimated based on Z-scores for each residue, and the results are shown beneath. The Z-score describes how many standard deviations the model quality is away from the average high-resolution X-ray structure. Negative values indicate that the homology model looks worse than a high-resolution X-ray structure. Quality Z-score: Dihedrals 0.884 (optimal), packing 1D/3D: -1.030 / -1.405 (satisfactory). The overall Z-scores have been calculated as the weighted averages of the individual Z-scores using the formula Overall = 0.145*Dihedrals + 0.390*Packing1D + 0.465*Packing3D. The overall Z-score of -0.927 indicates that the modeling procedure resulted in a good homology model. The position of the residue ASN251 is indicated by the arrow. Asn251 is located in an α-helical region which could be optimally modeled.

Figure S1

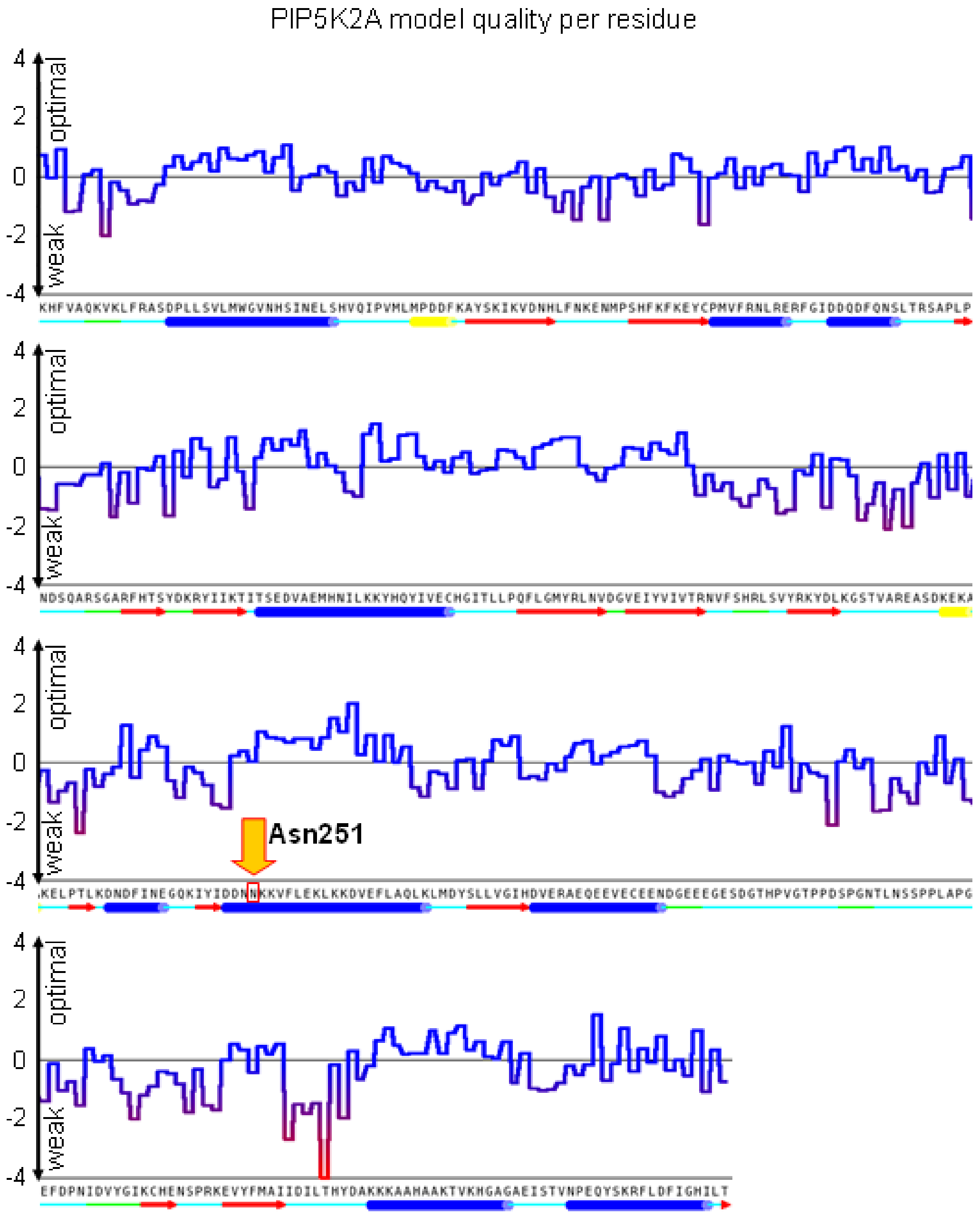

Suppl. Fig 1, Seebohm *et al.*

Supplemental Fig 2. Predicted secondary structure of a GluA1 peptide and quality of the GluA1 homology model. **a** The sequence of the generated partial GluA1 peptide LAMLVALIEFCYKSRSESKRMKG was submitted to the PSIPRED3.0 Server (Bryson et al, 2005) to predict the putative secondary structure of the peptide (Jones, 1999). Results indicated the formation of an α-helix along the whole peptide. **b** The final GluA1 homology model was subjected to a final round of simulated annealing minimization in explicit solvent using YASARA Structure v10.1. The quality check based on the Z-score provided the following values: dihedrals 0.946 (optimal), packing 1D/3D -0.995 (good) / -1.158 (satisfactory) and an overall Z-score (computed as in Suppl. Fig. 1) of - 0.789, suggesting an overall good homology model.

Figure S2

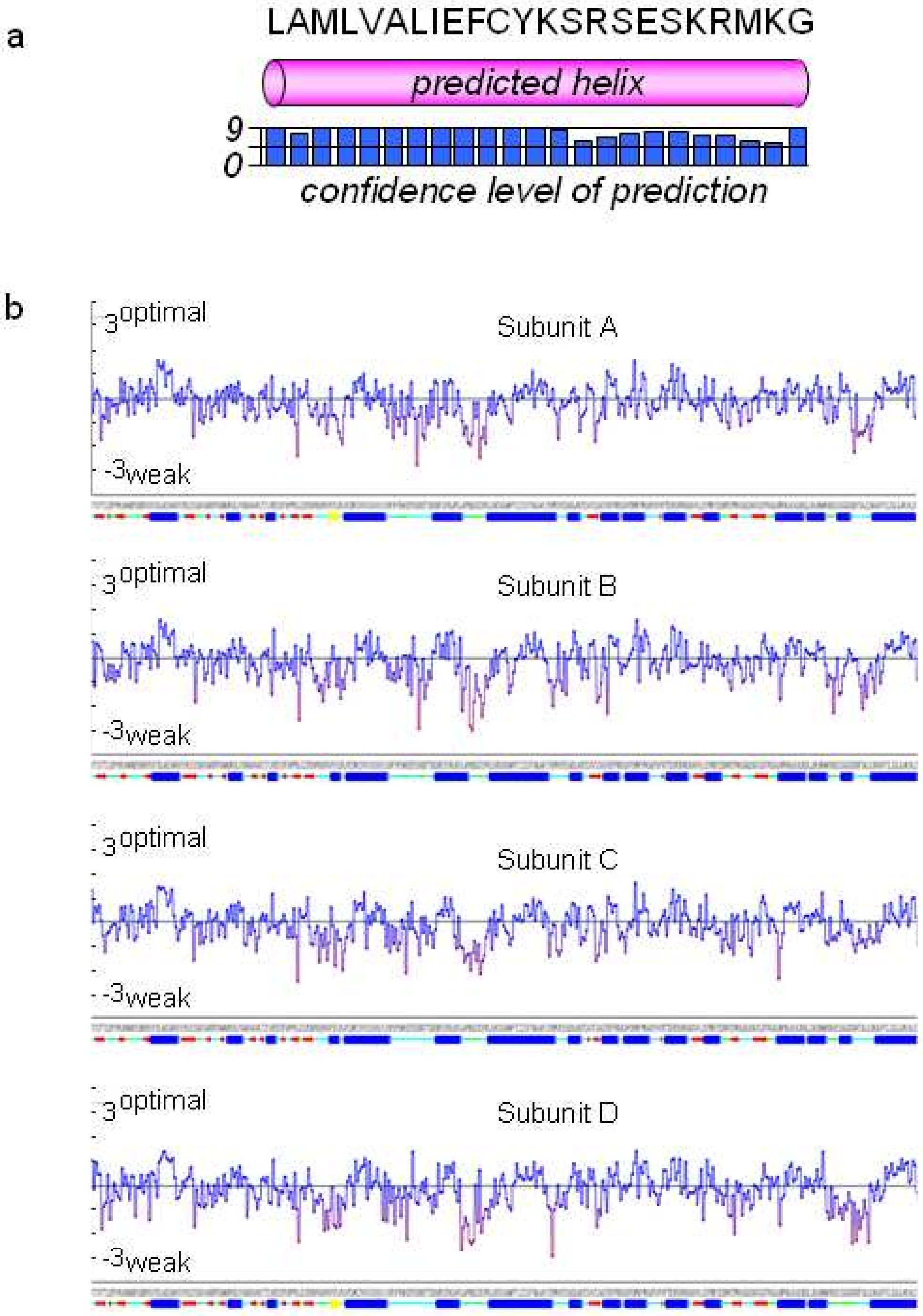

Suppl. Fig 2. Seebohm *et al.*

Supplemental Fig 3. Analyses of RMSF of PIP5K2A models. The Root Mean Square Fluctuations (RMSF) around average structures of the PIP5K2A wt and PIP5K2A(N251S) including respective ligands were analyzed. The black and the red lines indicate PIP5K2A wt and PIP5K2A(N251S), respectively. The data show that the mutation N251S has effects on several other regions in the models.

Figure S3

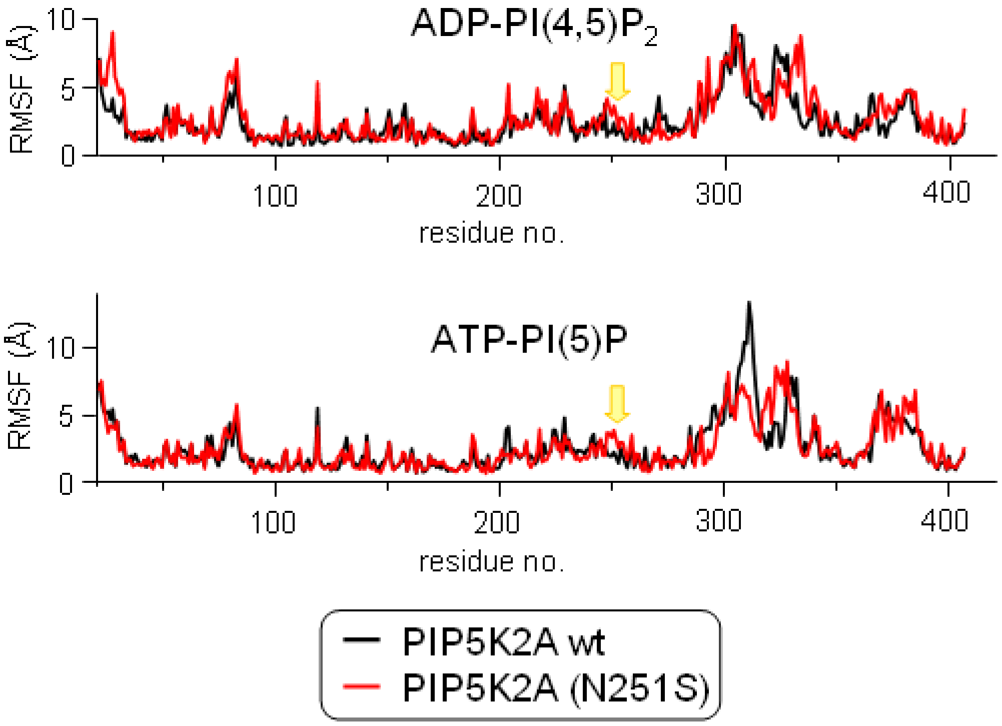

Suppl. Fig 3. Seebohm *et al.*

Supplemental Fig 4. Surface-rendered PIP5K2A models with ligands. The surface of average structures of the PIP5K2A wt (blue) and PIP5K2A(N251S) (red) were rendered. The bound ligands are shown in yellow (ATP/ADP and PI(5)P/PI(4,5)P_2 bound to PIP5K2A(N251S)) and turquoise (ATP/ADP and PI(5)P/ PI(4,5)P_2 bound to PIP5K2A wt). A prominent altered binding conformation of ligands in the PIP5K2A(N251S)-ATP-PI(5)P model compared to the PIP5K2A wt-ATP-PI(5)P model can be observed (upper models).

Figure S4

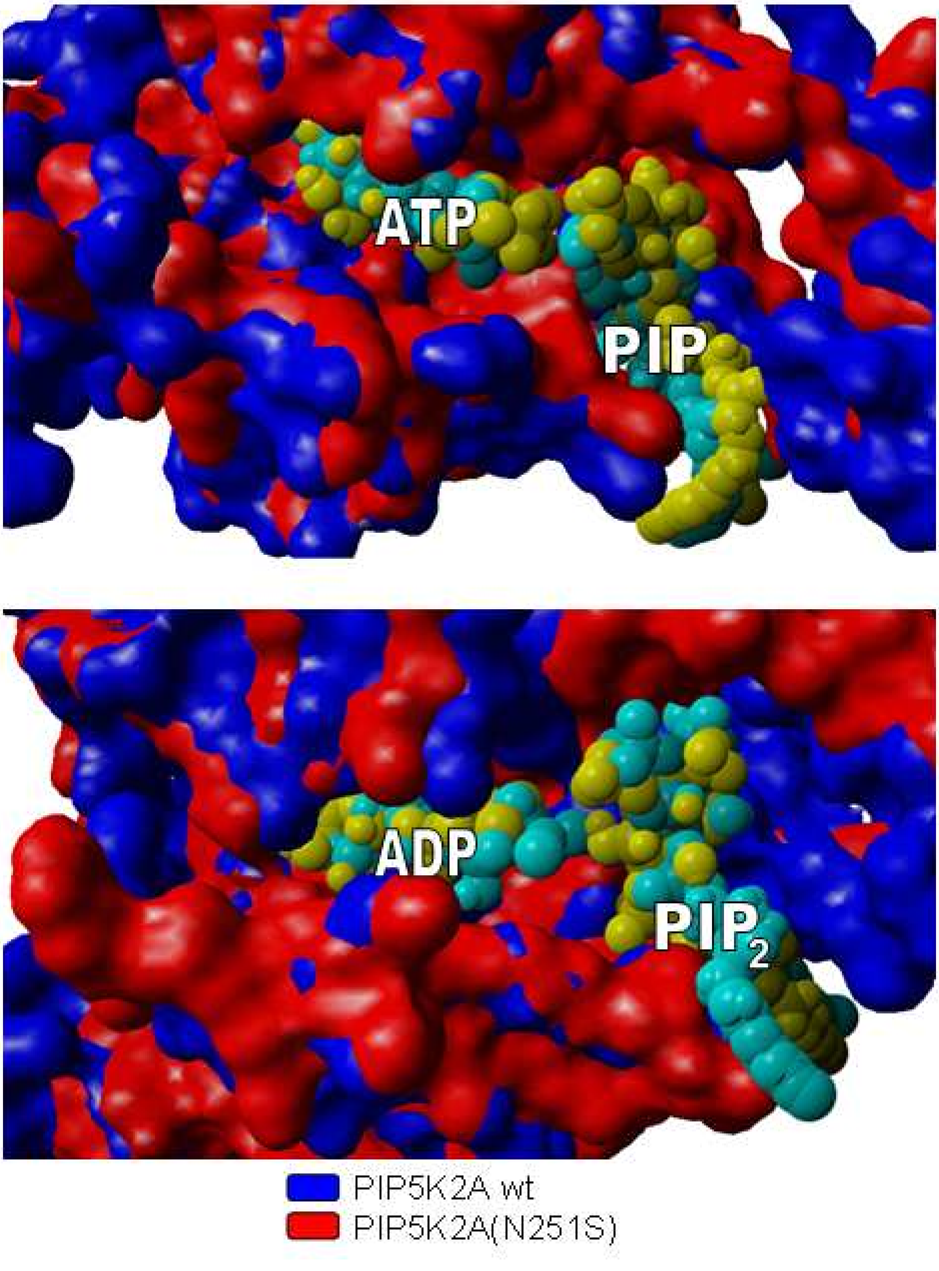

Suppl. Fig 4. Seebohm *et al.*

Supplemental Fig 5. Detailed analyses of GluA1 currents in HEK293 cells with or without water soluble PI(4,5)P_2 diC8 in the recording pipet in outside-out mode. Individual values, mean values and SEM of experiments in absence (control) or presence of PI(4,5)P_2 diC8 in the recording pipet. Peak amplitudes (± S.E.M.) of peak currents evoked by 10 ms application of 1 mM glutamate (mean peak values control: 22 ± 4 pA, +PIP_2 70 ± 11 pA; significance p = 0,0002, Mann-Whitney-test). To compensate for patches of different sizes, current densities were calculated (control: 32 ± 6 pA / pF, +PIP_2 diC8: 66 ± 13 pA / pF; significance p = 0,0262, Mann-Whitney-test). Time-to-peak values almost reached significance in p = 0,07, Mann-Whitney-test. Analysis of the time constants of desenzitation clearly showed no significant differences p= 0.7 in Mann-Whitney-test. To further analyse possible effects on desensitazation cyclothiacide, a compound inhibiting desensitazation, was applied. The ratio of peak current under CTZ (CTZ peak) and the sustained current (SS) was calculated. This analysis clearly showed no significant differences p= 0.9 in Mann-Whitney-test.

Figure S5

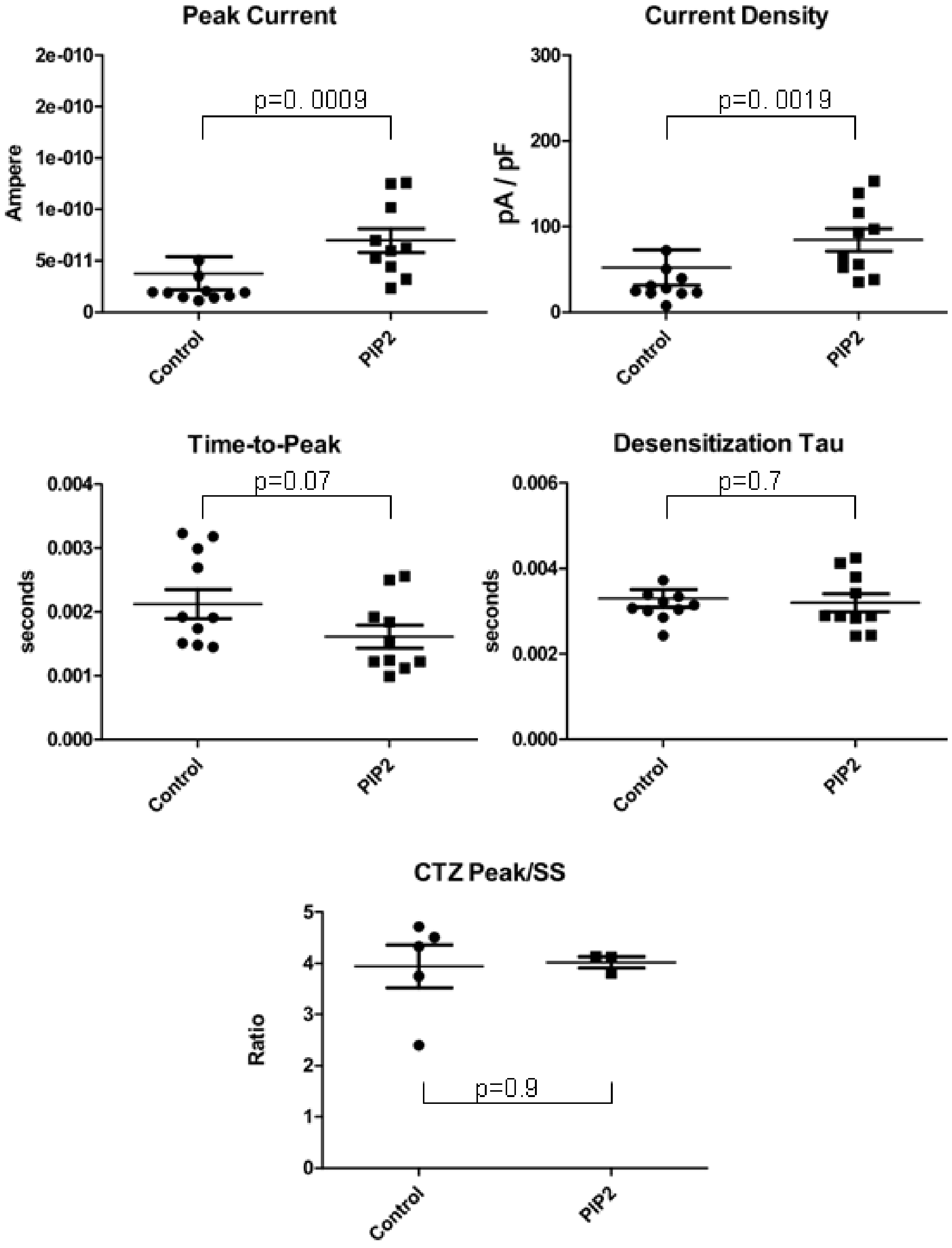

Suppl. Fig 5. *Seebohm et al.*

REFERENCES

Banke TG, Bowie D, Lee H, Huganir RL, Schousboe A, Traynelis SF (2000) Control of GluR1 AMPA receptor function by cAMP-dependent protein kinase. J Neurosci 20(1): 89-102

Bryson K, McGuffin LJ, Marsden RL, Ward JJ, Sodhi JS, Jones DT (2005) Protein structure prediction servers at University College London. Nucleic Acids Res 33(Web Server issue): W36-38

Hausser M, Roth A (1997) Dendritic and somatic glutamate receptor channels in rat cerebellar Purkinje cells. J Physiol 501 (Pt 1): 77-95

Heckmann M, Bufler J, Franke C, Dudel J (1996) Kinetics of homomeric GluR6 glutamate receptor channels. Biophys J 71(4): 1743-1750

Jonas P, Major G, Sakmann B (1993) Quantal components of unitary EPSCs at the mossy fibre synapse on CA3 pyramidal cells of rat hippocampus. J Physiol 472: 615-663

Jones DT (1999) Protein secondary structure prediction based on position-specific scoring matrices. J Mol Biol 292(2): 195-202
